# Simple Energy Balance or Microbiome for Childhood Obesity Prevention?

**DOI:** 10.3390/nu13082730

**Published:** 2021-08-09

**Authors:** Tom Baranowski, Kathleen J. Motil

**Affiliations:** USDA/ARS Children’s Nutrition Research Center, Department of Pediatrics, Baylor College of Medicine, 1100 Bates St, Houston, TX 77030, USA; kmotil@bcm.edu

**Keywords:** behavioral nutrition, microbiome, energy balance, children, obesity prevention, multietiological

## Abstract

Obesity prevention interventions generally have either not worked or had effects inadequate to mitigate the problem. They have been predicated on the simple energy balance model, which has been severely questioned by biological scientists. Numerous other etiological mechanisms have been proposed, including the intestinal microbiome, which has been related to childhood obesity in numerous ways. Public health research is needed in regard to diet and the microbiome, which hopefully will lead to effective child obesity prevention.

## 1. Introduction

Obesity is a modern health scourge, common among adults across 195 countries [1]. In the US, it is more common among lower income [2,3] and ethnic minority [4] individuals, and increasingly more common among children, with rates of increase higher than those in adults [1]. Obese children tend to become obese adults [5]. Only physical activity was strongly inversely related to obesity among preschool children [6]. Neither food groups [7] nor physical activity (PA) alone, or in combination [8], accounted for obesity in all other age groups. Thus, the causes of obesity need to be clearly delineated to enable effective childhood obesity prevention [9].

Any intervention that purports to influence a biological outcome must be predicated on a biological mechanism. This paper argues that simple energy balance has been shown to be inadequate to account for obesity. Within a multi-ecological approach, there are other biological mechanisms, e.g., the microbiome, circadian rhythms, adenovirus 36, or aspects of air pollution [9] that may impact obesity outcomes. Herein, we identify the limitations of the simple energy balance model and explore the microbiome as one among many etiological factors in childhood obesity. Influencing the microbiome to prevent childhood obesity would require behavior changes, but those would be quite different from those proposed by simple energy balance.

## 2. Discussion

Numerous systematic reviews have reported on childhood obesity prevention interventions. In the most comprehensive review of randomized controlled trials (RCTs) (*n* = 153 RCTs) [10], combined diet and PA interventions reduced body mass index (BMI) (mean difference = −0.07 kg/m^2^) and BMI z-score (BMIz) (mean difference = −0.11) among 0–5-year-olds, but the confidence intervals encompassed zero. Both changes were small and were obtained immediately post-intervention, suggesting no long-term effects [10]. In other age groups (6–12 years, 13–18 years), meta-analyses revealed few effects, and when obtained, these effects were small with an inconsistent pattern across intervention components (diet, PA, both) and age groups [10]. A review of the forest plots from a meta-analysis of school-based child obesity prevention interventions revealed that the vast majority of effects hovered near zero [11]. When reviewing the impact of all child obesity prevention trials on energy balance [12], only small, weak effects on energy consumed and energy expended were noted. Even longer term (one year or longer) trials did not impact BMI [13].

Most child obesity prevention trials did not have adequate financial resources to deliver a comprehensive intervention program, or had an inadequate sample size to test their effects [10,14]. Two of the largest well-financed childhood obesity prevention studies with multi-component (nutrition, PA, behavior modification) interventions, the HEALTHY (*n* = 4603 across 42 schools in seven US cities) [15] and IDEFICS (*n* = 16,228 from nine European counties) trials [16], demonstrated no significant effect on obesity. One report considered theories, targeted behaviors, intervention designs, and implementation. It also identified problems at each step in the chain of effects from intervention procedures to mediating variables, behavior change and health outcomes [14].

### 2.1. Models of Energy Balance

The concept of energy balance has dominated thinking about obesity prevention. The simple, also called static [17], energy balance model proposes that increased energy intake (e.g., from food calories) in excess of energy expenditure (e.g., from PA) is linearly related to increased body weight (i.e., fat storage); and increased energy expenditure (e.g., from exercise) in excess of energy intake is linearly related to weight loss [18]. This model was rejected for being too simple [18]. A major limitation is that it does not account for diverse feedback mechanisms including body size, weight adaptation, hunger, satiety [19], and the release of hormones, e.g., leptin, adiponectin, which impact hunger and satiety [20,21]. Thus, while the law of thermodynamics says that energy cannot be destroyed or created, the human body is not a simple mechanical motor efficiently converting energy intake to energy output. Factors internal to the body, e.g., hormones [20,21], influence the energy available and energy expenditure beyond simple dietary energy intake and expenditure. Inconsistencies in the simple energy balance model have become apparent. These inconsistencies include observations that: (a) some energy dense foods (e.g., olive oil, nuts) are not associated with weight gain (which may be due in part to energy bio-inaccessibility of some foods [22,23]); (b) some ethnic groups have a greater susceptibility to obesity [24]; and (c) the intake of indigestible fiber (i.e., no calorie intake) is associated with lower adiposity [25,26]. These observations question the usefulness of the simple energy balance model for understanding obesity prevention [27].

Models have been proposed to understand a more dynamic weight control process, including the control set point with its homeostatic and non-homeostatic influences [17,28]. With the elucidation of the importance of non-homeostatic influences [29], a settling point [30], and cognitive feedback mechanisms [31], including reward pathways activated by palatable food, self-control, and social influences [32], were proposed. In a metabolic tipping point model [33], energy expenditure across variable levels of PA and energy intake in adults were directly related, thereby maintaining a direct relationship with body mass, but at a low level of PA, energy expenditure and intake became dissociated and body mass increased [33]. Contrary to adults, in lean and obese children, there is an uncoupling of the relationship between energy expenditure and energy intake [34] across a range of PA levels. In all cases, simple energy balance was inadequate to account for the related data. Thus, a complex energy balance model relevant to weight control must be informed by diverse feedback mechanisms [34], and expanded to encompass related biological mechanisms.

### 2.2. Other Possible Causes of Childhood Obesity

A large number of possible causes of obesity have been identified [35], suggesting a multi-etiological genesis of obesity [9]. The extent to which genetics accounts for the variation in obesity in heritability studies varied from 40 to 50%, with higher heritability among those at the higher end of the BMI distribution [36]. Although single-gene causes of obesity have been identified, e.g., Prader–Willi syndrome [37], genome-wide association studies (GWAS) reveal that the combination of the most likely specific causal genes account for perhaps 3% of the variability in the common type of obesity [38]. Identical twin dyads fed the same calorie controlled diets, or exposed to the same caloric level of exercise, responded to substantially different extents both within and between diads [36], suggesting factors other than dietary or activity calories are at play. Epigenetics, which controls gene expression, has been offered as a cause of obesity [39,40], while others argued for the combination of genetic and epigenetic factors [41]. While interventions can be tailored to alternative values of a genetic variant, no single genetic variant has been shown to relate to a sufficient number of cases of obesity to make this worthwhile as a public health intervention.

Influences during the perinatal, infancy, and early childhood periods, sometimes called developmental programming [42], appear to influence childhood obesity in ways other than simple energy balance [43,44,45], including in utero exposure to famine [46], maternal prenatal weight [45], breastfeeding [47], numerous in utero exposures [44], antibiotic use early in life [48], dietary sugar intake [49], dietary intake of ultra-processed foods [50], infection with Adenovirus 36 [51,52,53], exposure to endocrine disruptors (e.g., chemicals in plastic bottles) [54], air pollution [55], food allergies [56], dysfunctional reward system in the orbitofrontal cortex [57,58], and even traditional medicinal plants [59]. The biological mechanisms by which each of these possible initiation factors influence the trajectory toward obesity are not clearly known, but must work through interrelationships with complex energy balance mechanisms. However, these biological influences have largely been ignored by behavioral scientists interested in obesity prevention.

In this context, a closer integration has been advocated for between biological and behavioral approaches to obesity research [60]. A multi-etiological approach to child obesity prevention has been proposed, which includes numerous known biological influences on obesity not usually considered by behavioral researchers [9,61,62]. Reconsidering the biological mechanism(s) of action underlying current child obesity prevention programs may provide novel and more effective ways forward. The microbiome provides one such possible mechanism [63]. Extensive research has revealed pervasive relationships between the microbiome, health in general [64], and obesity in particular [65]. Only a small number of studies on the microbiome have been performed among children, and so findings from adults, and occasionally mammals, are reported here to provide a fuller picture.

### 2.3. Microbiome

The microbiome consists of 10 to 100 trillion microorganisms (bacteria, fungi, viruses, archaea, protozoa, and eukaryotes) that live mostly in each person’s gastrointestinal tract [66]. The microbiome is important because it protects the host against pathogens, metabolizes dietary nutrients and drugs [67,68], induces the absorption and distribution of dietary components [69], and bi-directionally communicates between the gastrointestinal tract and the central nervous system (called the gut–brain axis) [70], which may contribute to depression and anxiety [71]. Several mechanisms have been proposed for how the microbiome might predispose to obesity (mostly from animal research), including: developmental programming [42], relative presence of polysaccharide metabolizing bacterial taxa [72], reciprocal relationship with bile acids [73], diet [74], the gut–brain axis [75], host gene expression [40,76], host inflammation and thermogenesis [77], and circadian rhythms [78]. How these possible influences relate to the complex energy balance model is not clear [79].

The dominant bacterial phyla, expressed as relative abundancy (%) in the gut of overweight/obese children, were *Firmicutes* (68%), *Actinobacteria* (24%), *Bacteroidites* (4%) and *Proteobacteria* (2%) [80]. Adults and children with obesity tended to have lower diversity of microbes and proportionally more *Firmicutes* to *Bacteroidites* (as a ratio) than non-obese people [65]. *Firmicutes* and other bacterial phyla found in the gut metabolize a form of dietary fiber (polysaccharides) that is otherwise indigestible, resulting in increased availability of short-chain fatty acids and monosaccharides. These substrates increase the available energy in the gut, thereby contributing to obesity [81]. *Bacteroidetes* bacteria in the gut break down plant starches and fibers, but are less efficient at metabolizing polysaccharides, thereby considered to be protective against obesity [81].

An increased microbial diversity manifested by increased richness has been related to a healthy microbiome [82] with a lower risk of obesity and related diseases [83]. Low richness has been associated with an increased risk of adiposity, insulin resistance, dyslipidemia, and inflammation [84]. Although it was thought that children with obesity had significantly more *Firmicutes* (16 times more than *Bacteroidites*) and less *Bifidobacteria* than lean children [80], conflicting results have been identified [85]. A meta-analysis of the composition of the microbiome found no relation between the ratio of *Firmicutes* to *Bacteroidetes* in the gut and obesity [86]; however, some individuals with obesity tended to have lower bacterial diversity [87]. Possible explanations for the conflicting results across studies include differences in the methods for determining the distribution across microbiota, lifestyle differences among study participants, geographic labeling conventions, and diets consumed, among other factors [88]. A large UK-US study reported that the abundance of 17 little studied microbial classes were consistently related to BMI in both countries, but relationships were stronger with visceral fat [89]. Furthermore, intestinal species were associated with indicators of a healthy diet, suggesting diets could be personalized to characteristics of the microbiome [89]. A Mendelian randomization analysis indicated both weighted median and genetic risk score analyses support for a causal relationship between an abundance of the *Lachnospiraceae* family and trunk fat mass [90].

Although definitive statements of causality may be premature [91], the evidence for a causal relationship between the microbiome and obesity comes from animal fecal transplant research [92]. Gut microbiota transplanted from obese mice to germ-free mice led to obesity in the formerly lean germ-free mice [93]. The reverse happened when microbiota from lean mice were transplanted to germ-free mice [93]. Similarly, microbiota transferred from lean versus obese humans to germ-free mice led to similar body types [94], with corresponding changes in the microbiota functioning in the mice [94]. Only one pilot randomized controlled trial (RCT) (*n* = 22) transplanted fecal microbiota from a human lean person to obese patients. Although a reduction in BMI did not occur, the 12-week time interval may have been too short, and the sample too small, to detect changes in BMI and the composition of their microbiome toward that of a lean person [95].

The relationships among the microbiome, obesity and diet are complex [96], and may vary by child age. The microbiome may be considered a partial mediating variable between diet and obesity: nutrients are metabolized by the microbiome, initiating any of the several mechanisms which in turn influence obesity and related physiological effects. Diet influences the composition of microbiota, with evidence that some of these differences are related to disease processes including obesity [65]. At least four microbiome-related mechanisms mediate the diet and obesity relationship, including: the metabolism of indigestible dietary fiber, resulting in the production of short-chain fatty acids, particularly butyrate [97], and bile acids that are anti-inflammatory and regulate carbohydrate and lipid metabolism [98]; the up-regulation of the gut endocannabinoid system tone which influences gut permeability and tissue adipogenesis [99]; the reduction in gut gene expression of fasting induced adipocyte factor (PNPLA3) which correlates inversely with BMI [100]; and the regulation of gut–brain axis crosstalk, which modulates peptide secretions (GLP-1, GLP-2, peptide YY) that influence appetite reduction [101]. Healthy young adults consuming a high-fat, low-fiber diet had an increased presence of bacteria that predispose to obesity and several chronic diseases [102]. Reducing dietary flavonoids (e.g., fruit, vegetables) among those with obesity reduced the metabolic activity of adipose tissue and energy expenditure [103]. These metabolic processes were reversed when flavonoids were added back to the diet. In one study, the low FODMAP diet (fermentable olio-, di-, mono-saccharides and polyols) which has been used successfully in treating irritable bowel syndrome, led to differences in the microbiome (e.g., lower *Bifidobacteria*) [104], and less obesity [105]. Consumption of different dietary sugars led to differences in the composition of the microbiome [106]. The consumption of probiotics (e.g., dairy products with live bacterial cultures) has been shown to influence body composition [107], but the role of the microbiome in that relationship is not clear [108]. The microbiome was more predictive of obesity than nutrients [109]. Prebiotics, i.e., a substrate selectively utilized by host microorganisms conferring a health benefit [110], including indigestible dietary fibers and polyphenols (e.g., green tea), favor the growth of specific microbial species and have been inversely associated with body weight in several studies [111]. A prebiotic enabled overweight/obese children to lose weight over 16 weeks [112], and the composition of the microbiome (a high *Prevotella* to *Bacteroides* ratio (P/B) compared to a low P/B ratio) predicted a 3.8 kg greater body fat loss, independent of the experimental conditions, in a weight loss trial [113]. Both a low fat and a low carbohydrate diet led to numerous changes in the microbiome composition at 3 months, but microbiome composition reverted to baseline at 12 months [114]. It is not clear if the reversion reflected some resistance of the microbiome to change, and thereby prelude to weight regain, or reflected the end of experimental control of the diet at three months [115].

Much interest exists in the interrelationships among maternal and child diet, microbiome, and body composition during the perinatal period. Mothers exposed to a high fat diet during gestation had infants with depleted *Bacteroides* microbes [116,117,118,119,120], specifically lower *Lactobacillus reuteri* and *Bifidobacterium* [116]. Conversely, a postpartum maternal diet low in fat influenced offspring bacterial colonization (e.g., lower *Bacteriodes*) [116]. Children of obese mothers had different distributions of microbiota compared with those of lean mothers, which may vary by socioeconomic status and a high fat diet (lower *Bacteroides*) [70]. The maternal microbiome influenced the neonate’s microbiome differently between vaginal and cesarean births, indicating that the method of delivery influences the neonate’s microbiome due the presence or absence of exposure to vaginal microbes. While breastfeeding directly reduces the risk of childhood obesity [121], this is likely mediated by breastfeeding’s impact on the child’s microbiome [70]. Similarly, the timing of formula and complementary feeding influences the microbiome and obesity [122,123]. For example, the earlier introduction of complementary feeding influenced 13 different types of bacteria at three months of life and 20 types at 12 months, resulting in higher levels of short-chain fatty acids at 12 months, increasing the likelihood of obesity [124]. An infant’s microbiome tends to evolve toward their adult microbiome by the end of the first year of life [125], reflecting their home’s social and physical environment, including food choices, suggesting that dietary interventions may be important early in life.

### 2.4. Gut–Brain Axis

The gut–brain axis has been investigated in relation to obesity [126,127]. The microbiome and the brain develop at the same time, thereby allowing for bidirectional influences. The microbiome influences the formation of neural circuits [128], neuron excitability [129], and eating behavior in relation to appetite-regulating hormones [130]. The small intestine is populated with large numbers of nerves that sense and respond to nutrients [131]. These nerves connect to various parts of the brain, particularly the hypothalamus, which controls satiety through the release of leptin, and to enteroendocrine cells in the stomach and duodenum, which control appetite through the release of ghrelin. Increased leptin increases satiety, while decreased ghrelin reduces appetite, both via receptor expression in the hypothalamus. Leptin is made primarily by adipocytes and enterocytes; the leptin receptor is expressed on many cell types, but is found primarily in the hypothalamus and hippocampus. The primary function of leptin is the regulation of adipose tissue mass. The activity of leptin is mediated through its inhibitory effects on hunger and stimulatory effects on satiety. In the hypothalamus, leptin counteracts neuropeptide Y, a hunger promoter secreted by neurons in the sympathetic system of the gut and in the hypothalamus [132]. Ghrelin is a hormone produced by enteroendocrine cells in the gastrointestinal tract, primarily the stomach; the ghrelin receptor is found in the hypothalamus and the anterior pituitary gland. The primary function of ghrelin is to stimulate appetite, thereby increasing food intake and storing fat. Ghrelin stimulates the hypothalamus and anterior pituitary, including neurons that produce neuropeptide Y, leading to increased food intake, particularly carbohydrates [133].

The microbiome influences the production of peptides that mimic appetite-regulating hormones [134]. In addition, short-chain fatty acids produced by *Firmicutes* (and other microbiota) not only increase the energy available to the host, but also influence the brain which in turn influences host metabolism, appetite, and food intake [135,136]. Some of the nerves extend to brown adipose tissue, activate sympathetic branches of the autonomic nervous system, and thereby likely increase energy expenditure without increasing physical activity [126]. How all these factors interrelate to the initiation and control of obesity is not clear, but must relate to the complex energy balance model [137].

A sociology of the microbiome is emerging wherein meta-sociological factors (e.g., urbanization in a global context) influence more immediate sociological and behavioral factors ((e.g., vaginal versus caesarean mode of infant delivery), dietary intake/nutrition (e.g., probiotics, prebiotics), infant feeding (e.g., breastfeeding and formula feeding), medical practices and medication uses (e.g., antibiotics)), which influence the microbiome and child health [138]. The microbiome has been demonstrated to have bidirectional effects with physical activity [139], which then needs to be accounted for in relation to obesity.

## 3. Implications for Research and Practice

Among numerous possible etiological influences on obesity [9], the microbiome has been suggested for the operationalization of personalized nutrition [140]. Key behavioral issues need to be explored, including how probiotics, indigestible fibers, and/or transplanted gut microbiota influence childhood obesity as mediated by changes in the microbiome. Behavioral interventions will be needed at the level of the child and family, including food preparation practices, to encourage children to consume probiotics and indigestible fibers. How satiety-enhancing foods, e.g., high-fat foods, influence the enteroendocrine release of leptin and ghrelin, and in turn influence appetite and obesity, needs to be determined. Within a multi-ecological approach to child obesity prevention, the microbiome offers promising mechanisms that deserve the attention of nutrition educators and behavioral/public health nutritionists.

## References

[1] Afshin A., Forouzanfar M.H., Reitsma M.B., Sur P., Estep K. (2017). Health effects of overweight and obesity in 195 countries over 25 years. N. Engl. J. Med..

[2] Wang Y., Beydoun M.A. (2007). The obesity epidemic in the United States-gender, age, socioeconomic, racial/ethnic, and geographic characteristics: A systematic review and meta-regression analysis. Epidemiol. Rev..

[3] Psaltopoulou T., Hatzis G., Papageorgiou N., Androulakis E., Briasoulis A., Tousoulis D. (2017). Socioeconomic status and risk factors for cardiovascular disease: Impact of dietary mediators. Hell. J. Cardiol..

[4] Jaacks L.M., Vandevijvere S., Pan A., McGowan C.J., Wallace C., Imamura F. (2019). The obesity transition: Stages of the global epidemic. Lancet Diabetes Endocrinol..

[5] Singh A.S., Mulder C., Twisk J.W., van Mechelen W., Chinapaw M.J. (2008). Tracking of childhood overweight into adulthood: A systematic review of the literature. Obes. Rev..

[6] te Velde S.J., van Nassau F., Uijtdewilligen L., van Stralen M.M., Cardon G., De Craemer M. (2012). Energy balance-related behaviours associated with overweight and obesity in preschool children: A systematic review of prospective studies. Obes. Rev..

[7] Schlesinger S., Neuenschwander M., Schwedhelm C., Hoffmann G., Bechthold A., Boeing H. (2019). Food groups and risk of overweight, obesity, and weight gain: A systematic review and dose-response meta-analysis of prospective studies. Adv. Nutr..

[8] Narciso J., Silva A.J., Rodrigues V., Monteiro M.J., Almeida A., Saavedra R. (2019). Behavioral, contextual and biological factors associated with obesity during adolescence: A systematic review. PLoS ONE.

[9] Baranowski T., Motil K.J., Moreno J.P. (2019). Multi-etiological perspective on child obesity prevention. Curr. Nutr. Rep..

[10] Brown T., Moore T.H., Hooper L., Gao Y., Zayegh A., Ijaz S. (2019). Interventions for preventing obesity in children. Cochrane Database Syst. Rev..

[11] Liu Z., Xu H.M., Wen L.M., Peng Y.Z., Lin L.Z., Zhou S. (2019). A systematic review and meta-analysis of the overall effects of school-based obesity prevention interventions and effect differences by intervention components. Int. J. Behav. Nutr. Phys. Act..

[12] Richardson A.S., Chen C., Sturm R., Azhar G., Miles J., Larkin J. (2019). Obesity prevention interventions and implications for energy balance in the United States and Mexico: A systematic review of the evidence and meta-analysis. Obesity.

[13] Cerrato-Carretero P., Roncero-Martín R., Pedrera-Zamorano J.D., López-Espuela F., Puerto-Parejo L.M., Sánchez-Fernández A. (2021). Long-term dietary and physical activity interventions in the school setting and their effects on BMI in children aged 6–12 years: Meta-analysis of randomized controlled clinical trials. Healthcare.

[14] Baranowski T., Lytle L. (2015). Should the IDEFICS outcomes have been expected?. Obes. Rev..

[15] Foster G.D., Linder B., Baranowski T., Cooper D.M., Goldberg L., Harrell J.S. (2010). A school-based intervention for diabetes risk reduction. N. Engl. J. Med..

[16] De Henauw S., Huybrechts I., De Bourdeaudhuij I., Bammann K., Barba G., Lissner L. (2015). Effects of a community-oriented obesity prevention programme on indicators of body fatness in preschool and primary school children. Main results from the IDEFICS study. Obes. Rev..

[17] Hall K.D., Guo J. (2017). Obesity energetics: Body weight regulation and the effects of diet composition. Gastroenterology.

[18] Ludwig D.S., Ebbeling C.B. (2018). The carbohydrate-insulin model of obesity: Beyond “calories in, calories out”. JAMA Intern. Med..

[19] Schoeller D.A. (2009). The energy balance equation: Looking back and looking forward are two very different views. Nutr. Rev..

[20] Neseliler S., Hu W., Larcher K., Zacchia M., Dadar M., Scala S.G. (2019). Neurocognitive and hormonal correlates of voluntary weight loss in humans. Cell Metab..

[21] Sumithran P., Prendergast L.A., Delbridge E., Purcell K., Shulkes A., Kriketos A. (2011). Long-term persistence of hormonal adaptations to weight loss. N. Engl. J. Med..

[22] Nishi S.K., Kendall C.W.C., Bazinet R.P., Hanley A.J., Comelli E.M., Jenkins D.J.A. (2021). Almond bioaccessibility in a randomized crossover trial: Is a calorie a calorie?. Mayo Clin. Proc..

[23] Salas-Salvadó J., Bulló M., Babio N., Martinez-Gonzalez M.A., Ibarrola-Jurado N., Basora J., Estruch R., Covas M.I., Corella D., Arós F. (2011). Reduction in the incidence of type 2 diabetes with the Mediterranean diet: Results of the PREDIMED-Reus nutrition intervention randomized trial. Diabetes Care.

[24] Franks P.W., Ravussin E., Hanson R.L., Harper I.T., Allison D.B., Knowler W.C., Tataranni P.A., Salbe A.D. (2005). Habitual physical activity in children: The role of genes and the environment. Am. J. Clin. Nutr..

[25] A Babiker R., Merghani T.H., Elmusharaf K., Badi R.M., Lang F., Saeed A.M. (2012). Effects of gum arabic ingestion on body mass index and body fat percentage in healthy adult females: Two-arm randomized, placebo controlled, double-blind trial. Nutr. J..

[26] Lim U., Monroe K.R., Buchthal S., Fan B., Cheng I., Kristal B.S., Lampe J.W., Hullar M.A., Franke A.A., Stram D.O. (2019). Propensity for intra-abdominal and hepatic adiposity varies among ethnic groups. Gastroenterology.

[27] Romieu I., Dossus L., Barquera S., Blottiere H., Franks P., Gunter M., Hwalla N., Hursting S.D., Leitzmann M., On behalf of the IARC working group on Energy Balance and Obesity (2017). Energy balance and obesity: What are the main drivers?. Cancer Causes Control..

[28] Yu Y.-H., Yi-Hao Y. (2017). Making sense of metabolic obesity and hedonic obesity. J. Diabetes.

[29] Borer K.T. (2010). Nonhomeostatic control of human appetite and physical activity in regulation of energy balance. Exerc. Sport Sci. Rev..

[30] Geary N. (2020). Control-theory models of body-weight regulation and body-weight-regulatory appetite. Appetite.

[31] Hall K.D., Hammond R.A., Rahmandad H. (2014). Dynamic interplay among homeostatic, hedonic, and cognitive feedback circuits regulating body weight. Am. J. Public Health.

[32] Bessesen D.H. (2011). Regulation of body weight: What is the regulated parameter?. Physiol. Behav..

[33] Archer E., Lavie C.J., Hill J.O. (2018). The contributions of ‘diet’, ‘genes’, and physical activity to the etiology of obesity: Contrary evidence and consilience. Prog. Cardiovasc. Dis..

[34] Thivel D., Chaput J.P. (2014). Are post-exercise appetite sensations and energy intake coupled in children and adolescents?. Sports Med..

[35] McAllister E.J., Dhurandhar N.V., Keith S.W., Aronne L.J., Barger J., Baskin M., Benca R.M., Biggio J., Boggiano M.M., Eisenmann J. (2009). Ten putative contributors to the obesity epidemic. Crit. Rev. Food Sci. Nutr..

[36] Bouchard C. (2021). Genetics of obesity: What we have learned over decades of research. Obesity.

[37] Khan M.J., Gerasimidis K., Edwards C.A., Shaikh M.G. (2018). Mechanisms of obesity in Prader-Willi syndrome. Pediatr. Obes..

[38] Müller M.J., Geisler C., Blundell J., Dulloo A., Schutz Y., Krawczak M., Bosy-Westphal A., Enderle J., Heymsfield S.B. (2018). The case of GWAS of obesity: Does body weight control play by the rules?. Int. J. Obes..

[39] Lai C.-Q., E Smith C., Parnell L.D., Lee Y.-C., Corella D., Hopkins P., A Hidalgo B., Aslibekyan S., A Province M., Absher D. (2018). Epigenomics and metabolomics reveal the mechanism of the APOA2-saturated fat intake interaction affecting obesity. Am. J. Clin. Nutr..

[40] Bonder M.J., Kurilshikov A., Tigchelaar E.F., Mujagic Z., Imhann F., Vila A.V., Deelen P., Vatanen T., Schirmer M., Smeekens S.P. (2016). The effect of host genetics on the gut microbiome. Nat. Genet..

[41] Diels S., Berghe W.V., Van Hul W. (2020). Insights into the multifactorial causation of obesity by integrated genetic and epigenetic analysis. Obes. Rev..

[42] Nguyen L.T., Pollock C.A., Saad S., Tappia P., Ramjiawan B., Dhalla N. (2020). The developmental mechanisms of obesity by maternal obesity. Pathophysiology of Obesity-Induced Health Complications.

[43] Symonds M.E. (2007). Integration of physiological and molecular mechanisms of the developmental origins of adult disease: New concepts and insights. Proc. Nutr. Acad.Soc. USA.

[44] Fernandez-Twinn D.S., Hjort L., Novakovic B., Ozanne S.E., Saffery R. (2019). Intrauterine programming of obesity and type 2 diabetes. Diabetologia.

[45] Heslehurst N., Vieira R., Akhter Z., Bailey H., Slack E., Ngongalah L., Pemu A., Rankin J. (2019). The association between maternal body mass index and child obesity: A systematic review and meta-analysis. PLoS Med..

[46] Jiang H., Yu Y., Li L., Xu W. (2021). Exposure to the great famine in early life and the risk of obesity in adulthood: A report based on the China health and nutrition survey. Nutrients.

[47] Marseglia L., Manti S., D’Angelo G., Cuppari C., Salpietro V., Filippelli M., Trovato A., Gitto E., Salpietro C., Arrigo T. (2015). Obesity and breastfeeding: The strength of association. Women Birth.

[48] Jess T., Morgen C.S., Harpsøe M.C., Sørensen T.I.A., Ajslev T.A., Antvorskov J.C., Allin K.H. (2019). Antibiotic use during pregnancy and childhood overweight: A population-based nationwide cohort study. Sci. Rep..

[49] Bentley R.A., Ruck D.J., Fouts H.N. (2020). U.S. obesity as delayed effect of excess sugar. Econ. Hum. Biol..

[50] Hall K.D., Ayuketah A., Brychta R., Cai H., Cassimatis T., Chen K., Chung S.T., Costa E., Courville A., Darcey V. (2019). Ultra-processed diets cause excess calorie intake and weight gain: An inpatient randomized controlled trial of ad libitum food intake. Cell Metab..

[51] Xu M.-Y., Cao B., Wang D.-F., Guo J.-H., Chen K.-L., Shi M., Yin J., Lu Q.-B. (2015). Human adenovirus 36 infection increased the risk of obesity: A meta-analysis update. Medicine.

[52] Kim J., Na H., Kim J.A., Nam J.H. (2020). What we know and what we need to know about adenovirus 36-induced obesity. Int. J. Obes..

[53] Fernandes J.D.S., Schuelter-Trevisol F., Cancelier A.C.L., e Silva H.C.G., de Sousa D.G., Atkinson R.L., Trevisol D.J. (2021). Adenovirus 36 prevalence and association with human obesity: A systematic review. Int. J. Obes..

[54] Rolfo A., Nuzzo A.M., De Amicis R., Moretti L., Bertoli S., Leone A. (2020). Fetal-maternal exposure to endocrine disruptors: Correlation with diet intake and pregnancy outcomes. Nutrients.

[55] Parasin N., Amnuaylojaroen T., Saokaew S. (2021). Effect of air pollution on obesity in children: A systematic review and meta-analysis. Children.

[56] Willis F.B., Shanmugam R., Southerland J.H., Mouton C.P. (2020). Food allergen elimination for obesity reduction; a longitudinal, case-control trial. Br. J. Gastroenterol..

[57] Pujol J., Blanco-Hinojo L., Martínez-Vilavella G., Deus J., Pérez-Sola V., Sunyer J. (2021). Dysfunctional brain reward system in child obesity. Cereb. Cortex.

[58] Rolls E.T. (2021). The orbitofrontal cortex, food reward, body weight, and obesity. Soc. Cogn. Affect. Neurosci..

[59] Sekhon-Loodu S., Rupasinghe H.P.V. (2019). Evaluation of antioxidant, antidiabetic and antiobesity potential of selected traditional medicinal plants. Front. Nutr..

[60] Kaiser K.A., Carson T.L., Dhurandhar E.J., Neumeier W.H., Cardel M.I. (2020). Biobehavioural approaches to prevention and treatment: A call for implementation science in obesity research. Obes. Sci. Pract..

[61] Valerio G., Bernasconi S. (2019). A multi-etiological model of childhood obesity: A new biobehavioral perspective for prevention?. Ital. J. Pediatr..

[62] Cardel M., Dulin-Keita A., Casazza K. (2011). Contributors to pediatric obesity in adolescence: More than just energy imbalance. Open Obes. J..

[63] Vyas N., Nair S., Rao M. (2019). Childhood obesity and diabetes: Role of probiotics and prebiotics. Global Perspectives on Childhood Obesity.

[64] Wu Z.A., Wang H.X.A. (2019). systematic review of the interaction between gut microbiota and host health from a symbiotic perspective. SN Compr. Clin. Med..

[65] Cornejo-Pareja I., Munoz-Garach A., Clemente-Postigo M., Tinahones F.J. (2019). Importance of gut microbiota in obesity. Eur. J. Clin. Nutr..

[66] Sender R., Fuchs S., Milo R. (2016). Revised estimates for the number of human and bacteria cells in the body. PLoS Biol..

[67] Javdan B., Lopez J.G., Chankhamjon P., Lee Y.-C.J., Hull R., Wu Q., Wang X., Chatterjee S., Donia M.S. (2020). Personalized mapping of drug metabolism by the human gut microbiome. Cell.

[68] Zimmermann M., Zimmermann-Kogadeeva M., Wegmann R., Goodman A.L. (2019). Mapping human microbiome drug metabolism by gut bacteria and their genes. Nature.

[69] Frame L.A., Costa E., Jackson S.A. (2020). Current explorations of nutrition and the gut microbiome: A comprehensive evaluation of the review literature. Nutr. Rev..

[70] Al Rubaye H., Adamson C.C., Jadavji N.M. (2021). The role of maternal diet on offspring gut microbiota development: A review. J. Neurosci. Res..

[71] Bear T.L.K., Dalziel J.E., Coad J., Roy N.C., Butts C.A., Gopal P.K. (2020). The role of the gut microbiota in dietary interventions for depression and anxiety. Adv. Nutr..

[72] Dao M.C., Everard A., Aron-Wisnewsky J., Sokolovska N., Prifti E., Verger E., Kayser B.D., Levenez F., Chilloux J., Hoyles L. (2016). Akkermansia muciniphila and improved metabolic health during a dietary intervention in obesity: Relationship with gut microbiome richness and ecology. Gut.

[73] Joyce S.A., Gahan C.G. (2016). Bile acid modifications at the microbe-host interface: Potential for nutraceutical and pharmaceutical interventions in host health. Annu. Rev. Food Sci. Technol..

[74] Carmody R.N., Gerber G.K., Luevano J.M., Gatti D.M., Somes L., Svenson K.L., Turnbaugh P.J. (2015). Diet dominates host genotype in shaping the murine gut microbiota. Cell Host Microbe.

[75] Bauer P.V., Hamr S.C., Duca F.A. (2016). Regulation of energy balance by a gut-brain axis and involvement of the gut microbiota. Cell Mol. Life Sci..

[76] Ussar S., Griffin N.W., Bezy O., Fujisaka S., Vienberg S., Softic S., Deng L., Bry L., Gordon J.I., Kahn C.R. (2015). Interactions between gut microbiota, host genetics and diet modulate the predisposition to obesity and metabolic syndrome. Cell Metab..

[77] Chassaing B., Ley R.E., Gewirtz A.T. (2014). Intestinal epithelial cell toll-like receptor 5 regulates the intestinal microbiota to prevent low-grade inflammation and metabolic syndrome in mice. Gastroenterology.

[78] Leone V., Gibbons S., Martinez K., Hutchison A.L., Huang E.Y., Cham C.M., Pierre J., Heneghan A.F., Nadimpalli A., Hubert N. (2015). Effects of diurnal variation of gut microbes and high-fat feeding on host circadian clock function and metabolism. Cell Host Microbe.

[79] Maruvada P., Leone V., Kaplan L.M., Chang E.B. (2017). The human microbiome and obesity: Moving beyond associations. Cell Host Microbe.

[80] Da Silva C.C., Monteil M.A., Davis E.M. (2020). Overweight and obesity in children are associated with an abundance of Firmicutes and reduction of Bifidobacterium in their gastrointestinal microbiota. Child. Obes..

[81] Riva A., Borgo F.C., Lassandro C., Verduci E., Morace G., Borghi E., Berry D. (2017). Pediatric obesity is associated with an altered gut microbiota and discordant shifts in Firmicutes populations. Environ. Microbiol..

[82] I McBurney M., Davis C., Fraser C.M., O Schneeman B., Huttenhower C., Verbeke K., Walter J., E Latulippe M. (2019). Establishing what constitutes a healthy human gut microbiome: State of the science, regulatory considerations, and future directions. J. Nutr..

[83] Cho I., Blaser M.J. (2012). The human microbiome: At the interface of health and disease. Nat. Rev. Genet..

[84] Le Chatelier E., Nielsen T., Qin J., Prifti E., Hildebrand F., Falony G., Almeida M., Arumugam M., Batto J.-M., Kennedy S. (2013). Richness of human gut microbiome correlates with metabolic markers. Nature.

[85] Lim Y.Y., Lee Y.S., Ooi D.S.Q. (2020). Engineering the gut microbiome for treatment of obesity: A review of current understanding and progress. Biotechnol. J..

[86] Sze M.A., Schloss P.D. (2016). Looking for a signal in the noise: Revisiting obesity and the microbiome. mBio.

[87] Cox L., Yamanishi S., Sohn J., Alekseyenko A., Leung J., Cho I., Kim G., Li H., Gao Z., Mahana D. (2014). Altering the intestinal microbiota during a critical developmental window has lasting metabolic consequences. Cell.

[88] Magne F., Gotteland M., Gauthier L., Zazueta A., Pesoa S., Navarrete P., Balamurugan R. (2020). The Firmicutes/Bacteroidetes ratio: A relevant marker of gut dysbiosis in obese patients?. Nutrients.

[89] Asnicar F., Berry S.E., Valdes A.M., Nguyen L.H., Piccinno G., Drew D.A., Leeming E., Gibson R., Le Roy C., Al Khatib H. (2021). Microbiome connections with host metabolism and habitual diet from 1098 deeply phenotyped individuals. Nat. Med..

[90] Xu Q., Zhang S.-S., Wang R.-R., Weng Y.-J., Cui X., Wei X.-T., Ni J.-J., Ren H.-G., Zhang L., Pei Y.-F. (2021). Mendelian randomization analysis reveals causal effects of the human gut microbiota on abdominal obesity. J. Nutr..

[91] Lynch K.E., Parke E.C., O’Malley M.A. (2019). How causal are microbiomes? A comparison with the Helicobacter pylori explanation of ulcers. Biol. Philos..

[92] Zhang Z., Mocanu V., Cai C., Dang J., Slater L., Deehan E.C., Walter J., Madsen K.L. (2019). Impact of fecal microbiota transplantation on obesity and metabolic syndrome-A systematic review. Nutrients.

[93] Turnbaugh P.J., Ley R.E., Mahowald M.A., Magrini V., Mardis E.R., Gordon J.I. (2006). An obesity-associated gut microbiome with increased capacity for energy harvest. Nature.

[94] Ridaura V.K., Faith J.J., Rey F.E., Cheng J., Duncan A.E., Kau A., Griffin N.W., Lombard V., Henrissat B., Bain J.R. (2013). Gut microbiota from twins discordant for obesity modulate metabolism in mice. Science.

[95] Allegretti J.R., Kassam Z., Mullish B., Chiang A., Carrellas M., Hurtado J., Marchesi J.R., McDonald J.A., Pechlivanis A., Barker G.F. (2020). Effects of fecal microbiota transplantation with oral capsules in obese patients. Clin. Gastroenterol. Hepatol..

[96] Vander Wyst K.B., Ortega-Santos C.P., Toffoli S.N., Lahti C.E., Whisner C.M. (2021). Diet, adiposity, and the gut microbiota from infancy to adolescence: A systematic review. Obes. Rev..

[97] Berni Canani R., Di Costanzo M., Leone L. (2012). The epigenetic effects of butyrate: Potential therapeutic implications for clinical practice. Clin. Epigenet..

[98] Harakeh S.M., Khan I., Kumosani T., Barbour E., Almasaudi S.B., Bahijri S.M., Alfadul S.M., Ajabnoor G.M.A., Azhar E.I. (2016). Gut microbiota: A contributing factor to obesity. Front. Cell. Infect. Microbiol..

[99] Muccioli G.G., Naslain D., Bäckhed F., Reigstad C.S., Lambert D.M., Delzenne N., Cani P.D. (2010). The endocannabinoid system links gut microbiota to adipogenesis. Mol. Syst. Biol..

[100] Wieser V., Adolph T.E., Enrich B., Moser P., Moschen A.R., Tilg H. (2017). Weight loss induced by bariatric surgery restores adipose tissue PNPLA3 expression. Liver Int..

[101] Everard A., Cani P.D. (2014). Gut microbiota and GLP-1. Rev. Endocr. Metab. Disord..

[102] Bailén M., Bressa C., Martínez-López S., González-Soltero R., Lominchar M.G.M., Juan C.S., Larrosa M. (2020). Microbiota features associated with a high-fat/low-fiber diet in healthy adults. Front. Nutr..

[103] Thaiss C.A., Itav S., Rothschild D., Meijer M., Levy M., Moresi C., Dohnalová L., Braverman S., Rozin S., Malitsky S. (2016). Persistent microbiome alterations modulate the rate of post-dieting weight regain. Nature.

[104] Zinocker M.K., Lindseth I.A. (2018). The Western diet-microbiome-host interaction and its role in metabolic disease. Nutrients.

[105] González M.S., Martín J.J.D., Treviño S.J., García C.A.B. (2019). Effect of healthy eating before intervention with a low FODMAP diet in pediatric patients with irritable bowel syndrome. Nutr. Hosp..

[106] Di Rienzi S.C., A Britton R. (2020). Adaptation of the gut microbiota to modern dietary sugars and sweeteners. Adv. Nutr..

[107] Borgeraas H., Johnson L.K., Skattebu J., Hertel J.K., Hjelmesaeth J. (2018). Effects of probiotics on body weight, body mass index, fat mass and fat percentage in subjects with overweight or obesity: A systematic review and meta-analysis of randomized controlled trials. Obes. Rev..

[108] Kristensen N.B., Bryrup T., Allin K.H., Nielsen T., Hansen T.H., Pedersen O. (2016). Alterations in fecal microbiota composition by probiotic supplementation in healthy adults: A systematic review of randomized controlled trials. Genome Med..

[109] Le Roy C.I., Bowyer R.C.E., Castillo-Fernandez J., Pallister T., Menni C., Steves C., Berry S.E., Spector T.D., Bell J.T. (2019). Dissecting the role of the gut microbiota and diet on visceral fat mass accumulation. Sci. Rep..

[110] Gibson G.R., Hutkins R., Sanders M.E., Prescott S.L., Reimer R.A., Salminen S.J., Scott K., Stanton C., Swanson K.S., Cani P.D. (2017). Expert consensus document: The International Scientific Association for Probiotics and Prebiotics (ISAPP) consensus statement on the definition and scope of prebiotics. Nat. Rev. Gastroenterol. Hepatol..

[111] Van Hul M., Cani P.D. (2019). Targeting carbohydrates and polyphenols for a healthy microbiome and healthy weight. Curr. Nutr. Rep..

[112] Nicolucci A.C., Hume M.P., Martinez I., Mayengbam S., Walter J., Reimer R.A. (2017). Prebiotics reduce body fat and alter intestinal microbiota in children who are overweight or with obesity. Gastroenterology.

[113] Hjorth M.F., Christensen L., Larsen T.M., Roager H.M., Krych L., Kot W., Nielsen D.S., Ritz C., Astrup A. (2020). Pretreatment Prevotella-to-Bacteroides ratio and salivary amylase gene copy number as prognostic markers for dietary weight loss. Am. J. Clin. Nutr..

[114] Fragiadakis G.K., Wastyk H.C., Robinson J.L., Sonnenburg E.D., Sonnenburg J.L., Gardner C.D. (2020). Long-term dietary intervention reveals resilience of the gut microbiota despite changes in diet and weight. Am. J. Clin. Nutr..

[115] Mueller N.T., Zhang M. (2020). Diet and long-term weight loss: What can we learn from our gut microbes?. Am. J. Clin. Nutr..

[116] Chu D.M., Antony K.M., Ma J., Prince A.L., Showalter L., Moller M. (2016). The early infant gut microbiome varies in association with a maternal high-fat diet. Genome Med..

[117] Bernardi J.R., Pinheiro T.V., Mueller N.T., Goldani H.A.S., Gutierrez M.R.P., Bettiol H., Da Silva A.A.M., Barbieri M.A., Goldani M.Z. (2015). Cesarean delivery and metabolic risk factors in young adults: A Brazilian birth cohort study. Am. J. Clin. Nutr..

[118] Mueller N., Shin H., Pizoni A., Werlang I.C., Matte U., Goldani M.Z., Goldani H.A.S., Dominguez-Bello M.G. (2016). Birth mode-dependent association between pre-pregnancy maternal weight status and the neonatal intestinal microbiome. Sci. Rep..

[119] Mueller N.T., Zhang M., Hoyo C., Østbye T., Benjamin-Neelon S.E. (2019). Does cesarean delivery impact infant weight gain and adiposity over the first year of life?. Int. J. Obes..

[120] Mueller N.T., Bakacs E., Combellick J., Grigoryan Z., Dominguez-Bello M.G. (2015). The infant microbiome development: Mom matters. Trends Mol. Med..

[121] Aguilar Cordero M.J., Sanchez Lopez A.M., Madrid Banos N., Mur Villar N., Exposito Ruiz M., Hermoso Rodriguez E. (2014). Breastfeeding for the prevention of overweight and obesity in children and teenagers; systematic review. Nutr. Hosp..

[122] Mohammadkhah A.I., Simpson E.B., Patterson S.G., Ferguson J.F. (2018). Development of the gut microbiome in children, and lifetime implications for obesity and cardiometabolic disease. Children.

[123] Gingras V., Aris I.M., Rifas-Shiman S.L., Switkowski K.M., Oken E., Hivert M.F. (2019). Timing of complementary feeding introduction and adiposity throughout childhood. Pediatrics.

[124] Differding M.K., Benjamin-Neelon S.E., Hoyo C., Ostbye T., Mueller N.T. (2020). Timing of complementary feeding is associated with gut microbiota diversity and composition and short chain fatty acid concentrations over the first year of life. BMC Microbiol..

[125] Bäckhed F., Roswall J., Peng Y., Feng Q., Jia H., Kovatcheva-Datchary P., Li Y., Xia Y., Xie H., Zhong H. (2015). Dynamics and stabilization of the human gut microbiome during the first year of life. Cell Host Microbe.

[126] Bliss E.S., Whiteside E. (2018). The gut-brain axis, the human gut microbiota and their integration in the development of obesity. Front. Physiol..

[127] Gupta A., Osadchiy V., Mayer E.A. (2020). Brain-gut-microbiome interactions in obesity and food addiction. Nat. Rev. Gastroenterol. Hepatol..

[128] Collins J., Borojevic R., Verdu E.F., Huizinga J.D., Ratcliffe E.M. (2014). Intestinal microbiota influence the early postnatal development of the enteric nervous system. Neurogastroenterol. Motil..

[129] McVey Neufeld K.A., Mao Y.K., Bienenstock J., Foster J.A., Kunze W.A. (2013). The microbiome is essential for normal gut intrinsic primary afferent neuron excitability in the mouse. Neurogastroenterol. Motil..

[130] Raybould H.E. (2010). Gut chemosensing: Interactions between gut endocrine cells and visceral afferents. Auton. Neurosci..

[131] Leeuwendaal N.K., Cryan J.F., Schellekens H. (2021). Gut peptides and the microbiome: Focus on ghrelin. Curr. Opin. Endocrinol. Diabetes Obes..

[132] Erlanson-Albertsson C. (2005). Appetite regulation and energy balance. Acta Paediatr..

[133] Horner K., Lee S. (2015). Appetite-related peptides in childhood and adolescence: Role of ghrelin, PYY, and GLP-1. Appl. Physiol. Nutr. Metab..

[134] Alcock J., Maley C.C., Aktipis C.A. (2014). Is eating behavior manipulated by the gastrointestinal microbiota? Evolutionary pressures and potential mechanisms. Bioessays.

[135] Fetissov S.O. (2017). Role of the gut microbiota in host appetite control: Bacterial growth to animal feeding behaviour. Nat. Rev. Endocrinol..

[136] Sandhu K.V., Sherwin E., Schellekens H., Stanton C., Dinan T.G., Cryan J.F. (2017). Feeding the microbiota-gut-brain axis: Diet, microbiome, and neuropsychiatry. Transl. Res..

[137] Torres-Fuentes C., Schellekens H., Dinan T.G., Cryan J.F. (2017). The microbiota-gut-brain axis in obesity. Lancet Gastroenterol. Hepatol..

[138] Brushett S., Sinha T., Reijneveld S.A., de Kroon M.L.A., Zhernakova A. (2020). The effects of urbanization on the infant gut microbiota and health outcomes. Front. Pediatr..

[139] Clauss M., Gérard P., Mosca A., Leclerc M. (2021). Interplay between exercise and gut microbiome in the context of human health and performance. Front. Nutr..

[140] Kolodziejczyk A.A., Zheng D., Elinav E. (2019). Diet-microbiota interactions and personalized nutrition. Nat. Rev. Microbiol..

